# Frequency of False Positive Rapid HIV Serologic Tests in African Men and Women Receiving PrEP for HIV Prevention: Implications for Programmatic Roll-Out of Biomedical Interventions

**DOI:** 10.1371/journal.pone.0123005

**Published:** 2015-04-17

**Authors:** Patrick Ndase, Connie Celum, Lara Kidoguchi, Allan Ronald, Kenneth H. Fife, Elizabeth Bukusi, Deborah Donnell, Jared M. Baeten, for the Partners PrEP Study Team

**Affiliations:** 1 Department of Global Health, University of Washington, Seattle, United States of America; 2 Department of Medicine, University of Washington, Seattle, United States of America; 3 Department of Epidemiology, University of Washington, Seattle, United States of America; 4 Department of Medicine, University of Manitoba, Winnipeg, Canada; 5 Infectious Disease Institute, Makerere University, Kampala, Uganda; 6 Department of Medicine, Indiana University, Indianapolis, United States of America; 7 Department of Obstetrics and Gynecology, University of Nairobi and Kenyatta National Hospital, Nairobi, Kenya; 8 Center for Microbiology Research, Kenya Medical Research Institute, Nairobi, Kenya; 9 Department of Obstetrics, Gynecology and Reproductive Sciences, University of California San Francisco, San Francisco, United States of America; 10 Statistical Center for HIV/AIDS Research and Prevention, Fred Hutchinson Cancer Research Center, Seattle, United States of America; Centers for Disease Control and Prevention, UNITED STATES

## Abstract

**Background:**

Rapid HIV assays are the mainstay of HIV testing globally. Delivery of effective biomedical HIV prevention strategies such as antiretroviral pre-exposure prophylaxis (PrEP) requires periodic HIV testing. Because rapid tests have high (>95%) but imperfect specificity, they are expected to generate some false positive results.

**Methods:**

We assessed the frequency of true and false positive rapid results in the Partners PrEP Study, a randomized, placebo-controlled trial of PrEP. HIV testing was performed monthly using 2 rapid tests done in parallel with HIV enzyme immunoassay (EIA) confirmation following all positive rapid tests.

**Results:**

A total of 99,009 monthly HIV tests were performed; 98,743 (99.7%) were dual-rapid HIV negative. Of the 266 visits with ≥1 positive rapid result, 99 (37.2%) had confirmatory positive EIA results (true positives), 155 (58.3%) had negative EIA results (false positives), and 12 (4.5%) had discordant EIA results. In the active PrEP arms, over two-thirds of visits with positive rapid test results were false positive results (69.2%, 110 of 159), although false positive results occurred at <1% (110/65,945) of total visits.

**Conclusions:**

When HIV prevalence or incidence is low due to effective HIV prevention interventions, rapid HIV tests result in a high number of false relative to true positive results, although the absolute number of false results will be low. Program roll-out for effective interventions should plan for quality assurance of HIV testing, mechanisms for confirmatory HIV testing, and counseling strategies for persons with positive rapid test results.

## Introduction

The use of antiretroviral-based approaches to HIV prevention, including antiretroviral treatment of HIV-infected partners of HIV-uninfected individuals [1], antiretroviral-based topical microbicides [2], and oral antiretrovirals as pre-exposure prophylaxis (PrEP) [3–6], has significant promise for reducing the scale of the HIV epidemic. Implementation of these HIV prevention interventions will be accompanied by periodic HIV testing; specifically for PrEP, testing is a critical step for continued usage of the intervention, since ongoing PrEP exposure with incident HIV infection may facilitate resistance.

In many settings, rapid HIV assays are the mainstay for HIV testing, with a number of developing countries recommending rapid testing according to a parallel or serial algorithms [7]. In the case of positive rapid results, or to clarify discordant or indeterminate rapid results, additional testing using either HIV enzyme immunoassay (EIA), Western blot, or RNA or DNA PCR may be done. HIV rapid test kits, although commonly having high sensitivity and specificity, generate false positive results since specificity is not 100 percent. [8–10]

As prevention interventions reduce HIV incidence, the proportion of HIV tests that are falsely positive, relative to truly positive, will rise, since effective prevention efforts will result in fewer individuals testing truly positive for HIV and rapid tests like all other HIV tests have an intrinsic rate of false positivity. Therefore, recognizing the potential for and frequency of false positive HIV rapid tests will become important for monitoring trends in HIV infection and also as a measure of success of HIV prevention interventions as they are rolled out. To investigate the rate of false-positive HIV rapid test results in the context of declining HIV incidence as a result of effective HIV prevention, we examined the frequency of positive rapid test results—both true and false—among persons followed prospectively in a clinical trial of PrEP for HIV prevention, in which parallel rapid tests were conducted followed by EIA confirmatory testing.

## Methods

### Study population

Data were from the Partners PrEP Study, a randomized, double-blind, placebo-controlled trial of once-daily oral tenofovir disoproxil fumarate (TDF) and emtricitabine (FTC)/TDF PrEP for HIV prevention among 4,747 heterosexual HIV serodiscordant couples from Kenya and Uganda. [3] The primary aims of the study were to determine the efficacy and safety of TDF and FTC/TDF PrEP, each compared to placebo, for prevention of HIV acquisition, and the design, methods, and results have been described previously. [3] In July 2011, the trial’s independent Data and Safety Monitoring Board recommended discontinuation of the placebo arm and public reporting of the results due to demonstration of efficacy for HIV protection for both TDF and FTC/TDF.

### Procedures

For HIV seronegative partners, trial eligibility criteria included normal renal function and being not pregnant or breastfeeding. At enrollment, HIV seronegative partners were assigned in a 1:1:1 ratio to one of three study arms: once-daily TDF, FTC/TDF, or placebo. HIV seronegative participants had monthly visits for up to 36 months, which included monthly HIV testing in order to promptly detect incident infections. Monthly visits also included dispensation of study medication, standardized assessment of sexual behavior, and clinical and laboratory safety monitoring.

### HIV testing algorithm

HIV testing was done monthly using two different rapid tests done in parallel with confirmatory testing using one HIV EIA when both rapid tests were positive and two different EIAs when only one of the rapid tests was positive. Thus the gold standard defining a true positive was either a) two positive rapids and a single positive EIA or b) a single positive rapid and two positive EIAs. In addition, for the present analysis, the gold standard defining a true negative was either a) two concordant negative rapid tests or b) a negative EIA result following a positive rapid test. Study sites used Determine paired with either Unigold or Bioline or Statpak, as per country-specific approved HIV testing (Table 1). All study sites were enrolled in an external quality assurance program for both HIV rapids and EIA. The EQA program applied to all HIV rapid test kits, and EIA. The EQA program supplier was the National Health Laboratory Services based in South Africa for HIV rapid and EIA. The frequency of EQA panel testing and reviews was three times (quarterly) per annum. All samples were blinded and sites performed them as per site specific SOP.

**Table 1:** HIV assays used, by study site.

	Kenya				Uganda				
	Eldoret	Kisumu	Nairobi	Thika	Jinja	Kampala	Kabwohe	Mbale	Tororo
**HIV-1 rapids**	Determine HIV 1/2 (Abbott /Inverness Medical) Manufacturer: JAPAN				Determine HIV 1/2 (Abbott /Inverness Medical) Manufacturer: JAPAN				
	Unigold (Trinity Biotech, USA)		Bioline (Standard Diagnostics, South Korea)		Unigold (Trinity Biotech, USA)			HIV 1/2 STAT-PAK (Chembio Diagnostic Systems, USA)	
**HIV-1 enzyme immunoassays**	Vironostika HIV Ag/Ab 4^th^ gen (bioMérieux, France)				Vironostika HIV Ag/Ab 4^th^ gen(bioMérieux, France)		Vironostika HIV Uni-Form II plus O – 3^rd^ gen (bioMérieux, France)		
	Murex HIV Ag/AB Combo 4^th^ gen (Abbott Murex, Ireland)				BioRad HIV 1/2 3^rd^ gen sold in Europe (Bio-Rad Laboratories, France)			Murex HIV 1.2.0 AB 3^rd^ gen (Abbott Murex, Ireland)	

Samples from all individuals who were confirmed HIV seropositive by HIV EIA at the study sites were further tested by HIV-1 Western Blot (BioRad Laboratories, Redmond, WA, USA) and HIV-1 RNA PCR (Abbott Real-time HIV-1, Abbott Molecular Inc., Des Plaines, IL, USA) at the University of Washington and were adjudicated by an HIV endpoints committee blinded to the randomization arm.

### Standard HIV prevention services

All participants received a comprehensive package of HIV prevention services at each monthly visit including: individual HIV-1 testing with pre- and post-test counseling, couples risk-reduction counseling, screening and treatment for sexually transmitted infections, free condoms with training and counseling, initiation of antiretroviral therapy for HIV-infected partners according to national guidelines, and referral for male circumcision and post-exposure prophylaxis according to national policies.

### Ethics Statement

The study protocol was approved by the University of Washington Human Subjects Review Committee and ethics review committees at each of the study sites, specifically the Moi University Institutional Research and Ethics Committee and Indiana University Human Subjects Office (Eldoret, Kenya site), Office of Research Administration, Kenya Medical Research Institute Ethics Review Committee and the University of California—San Francisco Committee for Human Research (Kisumu, Kenya site), Kenyatta National Hospital Ethics and Research Committee (Nairobi and Thika, Kenya sites), the National HIV/AIDS Research Committee of the Uganda National Council for Science and Technology (Jinja, Kabwohe, and Kampala, Uganda sites), and the Uganda Virus Research Institute Science and Ethics Committee and the Centers for Disease Control and Prevention Human Research Protection Office (Mbale and Tororo, Uganda sites). All participants provided written informed consent in English or their local language; the consent process and documents were approved by the overseeing ethics committees.

### Statistical analysis

The present analysis includes data through 10 July 2011. All study months with HIV rapid test results were included. For a small number of months (n = 361) in which more than one set of tests was done, the first was selected. Descriptive statistics—i.e., medians and percentages—were calculated. To determine if false positives were correlated with timing within the study (i.e., if false positives were greatest soon after each site initiated the trial and declined thereafter), we used a generalized estimating equation model to test for a relationship between months since the first enrollment at each site and false positive frequency. All analyses were done using SAS version 9.4 (SAS, Cary, North Carolina, USA).

## Results

Of the 4747 initially HIV seronegative participants enrolled in the trial, a total of 4722 (99.5%) had at least one follow-up HIV test during the study. The median age was 33.5 years (interquartile range [IQR] 28–40), and 2941 (62%) were male. Over a median follow-up period of 23 months (IQR 16 to 28), HIV incidence was 1.3% per year overall: <1% for the active PrEP arms (0.9% TDF and 0.6% FTC/TDF) and 2.3% in the placebo arm.

During 99,009 study months of follow-up, a total of 198,018 rapid HIV tests were performed in paired testing (Table 2). 266 of 99,009 months (0.3%) had ≥1 positive rapid test result: 99 of these 266 months (37.2%) had confirmatory positive EIA results, 155 of 266 months (58.3%) had a negative confirmatory EIA, thus indicating false positive rapid results, and 12 of 266 months (4.5%) had discordant EIA results (i.e., when two EIA tests were run following only a single positive rapid test and the results between the EIA tests were discrepant). Thus, in total, 163 individual tests (147 from discordant rapids and 8 pairs of concordant positives) from 155 study months were falsely positive. Sixty subjects (1.3% of the study cohort) had at least one visit with falsely positive rapid test results, of whom 23 had multiple visits with false positive results; of the 155 study months with false positive results, 95 (61.3%) occurred in individuals who had previously had a month with a false positive result.

**Table 2:** HIV test results.

	HIV testing result, # of study months**							
		Total	Both rapids negative	Both rapids positive, EIA negative	Both rapids positive, EIA positive*	Rapid tests discordant, Both EIAs negative	Rapid tests discordant, Both EIAs positive*	Rapid tests discordant, EIAs discordant
**Trial randomization arm**	**TDF**	32,837	32,753	0	17	57	6	4
	**FTC/TDF**	33,108	33,033	2	11	51	6	5
	**Placebo**	33,064	32,957	6	48	39	11	3
	**Total**	99,009	98,743	8	76	147	23	12

* Of the 99 cases with either one or two positive rapids followed with a positive EIA result (99 = 76+23), 96 (97.0%) were confirmed by centralized HIV Western blot and RNA testing; three results did not confirm.

** A single EIA was performed when both rapids are positive, two EIAs were performed when rapids are discordant.

False positive results were less likely to occur if both rapid tests were positive (9.5%, 8/84 months) versus if only one of the two rapid tests was positive (80.8%, 147/182 months). Each type of rapid test used had a <0.5% false positive frequency in this setting: 72/99,009 (0.07%) for Determine test kits (54/65,945, 0.08% for those receiving PrEP and 18/33,064, 0.05% for those receiving placebo); 18/53,124 for Unigold test kits (0.03%) (13/35,369, 0.04% for those receiving PrEP and 5/17,755, 0.03% for those receiving placebo); 3/19,277 (0.02%) for Bioline test kits (1/12,779, <0.01% for those receiving PrEP and 2/6,498, 0.03% for those receiving placebo); and 69/26,608 (0.26%) for Stat-Pak test kits (43/17,797, 0.24% for those receiving PrEP and 26/8,811, 0.30% for those receiving placebo); for one falsely positive result, the positive test kit was not known. The frequency of false positive tests was <0.5% of visits for each study site. There was no statistically significant relationship between duration of time a study site had been enrolling participants and the frequency of false positive results (p = 0.5).

Overall, in the active PrEP arms, more than two-thirds of visits with positive test results were false positives (69.2%, 110 of 159).

## Discussion

In this prospective HIV prevention study conducted in Kenya and Uganda, the rate of false positive rapid test results, although low (0.2%) relative to total number of tests done and within the expected frequency (<0.5%) for the rapid tests used, illustrates a potentially emerging challenge in HIV testing and prevention. Among those receiving PrEP for HIV prevention, which lowered HIV incidence to <1% per year (<0.1% per month), the number of false positive results (110) was greater than the number of true positive results (59).

The phrase ‘false positive paradox’ describes our primary findings. When the incidence of any given condition is lower than a test's false positive rate, even tests that have a high specificity (i.e., low chance of giving a false positive in an individual case) will generate more false than true positives. Our data illustrate this particularly for the active PrEP arms. Therefore, as HIV prevention activities in at-risk populations are successfully implemented, true positive HIV results should decline, as the number of incident HIV infections decline, but false positives will occur as they are inherent to the testing assay itself and are not influenced by prevention efforts.

According to WHO, the minimum required specificity and sensitivity for an HIV rapid test is 98% and 99%, respectively [7] and our results demonstrated specificity >99% for each of the test brands we used. In one study from South Africa, however, sensitivity and specificity were found to be 93–97% and 97–98%, respectively, for a number of different HIV rapid test assays assessed [11]. Further reduced assay performance has been seen in studies evaluating the performance of rapid tests in field settings, compared with laboratory settings [12]; thus, in field settings, a higher number of false positive results would be anticipated to arise as a result of poor test execution.

HIV testing algorithms based on rapid tests commonly incorporate strategies to improve specificity, including serial tests or confirmatory tests such as EIA, Western blot, or PCR. Unlike most national algorithms in Africa, which use serial rapid tests, our study used two rapid tests in parallel, which was done to maximize sensitivity to detect HIV infection early in the context of a PrEP clinical trial. Nonetheless, the principles of our findings are relevant to different rapid test algorithms, and the HIV rapid test kits used in this study are used serially in national programs.

This study had limitations. First, the results come from a phase III randomized trial, with substantial attention to laboratory oversight and ongoing external quality assurance; thus, the test performance was optimized and higher rates of false results might be expected to occur in typical field settings. Second, the trial protocol’s HIV testing algorithm used two different rapid test kits in parallel, unlike most national testing programs where serial HIV rapid tests are run. We thus may have ended up with a higher number of discordant HIV rapid test results than would be found in national programs, where a second different rapid HIV test kit would be performed only if the initial test kit result were to be positive. Finally, we did not attempt to assess the sensitivity of the rapid tests used in our study, as to do so would have required assessing all 99,000 visits by a gold standard sensitive HIV assay; our goal was to assess specificity only, particularly the impact of less than perfect specificity on the comparative rates of false and true positive results.

As implementation of effective biomedical HIV prevention interventions are implemented in field settings, particularly highly-effective strategies such as ART and PrEP, periodic HIV testing using HIV rapid tests will become central to monitoring success of these interventions, on both the individual and population level. HIV incidence will decrease and the proportion of positive rapid HIV tests that will be false positives will increase. Program evaluation for effective interventions such as PrEP should thus plan for quality assurance of HIV testing, mechanisms for confirmatory HIV testing, and counseling strategies for persons with positive rapid test results. Efficient and quality HIV testing mechanisms are thus an essential component of the success of scale up of HIV prevention.
